# A Systematic Review of the Orthopaedic Literature on Truncal and Lower Extremity Injuries in Major League Baseball Players

**DOI:** 10.5435/JAAOSGlobal-D-21-00030

**Published:** 2021-08-03

**Authors:** Hasani W. Swindell, Josephine R. Coury, Nicholas Dantzker, Cesar D. Lopez, Bryan M. Saltzman, David P. Trofa, Christopher S. Ahmad

**Affiliations:** From the Department of Orthopedics, Columbia University Medical Center, New York, NY (Dr. Swindell, Dr. Coury, Dr. Dantzker, Lopez, Dr. Trofa, Dr. Ahmad), and the Department of Orthopaedic Surgery, Atrium Health Musculoskeletal Institute, Charlotte, NC (Dr. Saltzman).

## Abstract

Orthopaedic injuries in Major League Baseball (MLB) players can have a tremendous impact on player health, longevity, salaries, and time lost from play.

**Purpose:**

To summarize all data published between January 1980 and August 2019 on truncal and lower extremity orthopaedic injuries sustained by MLB players.

**Methods:**

A literature review of studies examining injuries in MLB was performed using the PubMed and Embase databases. Included studies focused on truncal and lower extremity injuries in professional baseball players. Studies pertaining to nonorthopaedic injuries, and case reports, were excluded.

**Results:**

A total of 41 articles met the inclusion criteria and were selected for the final analysis. Articles were divided based on anatomic region of injury: hip and pelvis (16%), thigh (15%), truncal (14%), knee (13%), and ankle (11%). Most studies (83.7%) were level 3 evidence. Most studies obtained data using publicly available internet resources (29.8%) compared with the MLB Health and Injury Tracking System (22.1%).

**Conclusion:**

This review provides physicians with a single source of the most current literature regarding truncal and lower extremity orthopaedic injuries in MLB players. Most research was published on hip and pelvic, truncal, and thigh injuries and consisted of level III evidence.

Major League Baseball (MLB) remains one of the most popular sport in the United States, reporting an average yearly revenue of almost $10 billion and viewership of nearly 14 million people for the 2019 World Series.^1^ Beyond simple viewership, participation in fantasy baseball among MLB fans has markedly increased in recent years, driving public demand for more in-depth and accessible information on individual player performance, advanced metrics, and player injuries, with a focus on the prognostic implications that these injuries might have on future return to play (RTP) and ultimate athletic performance of the affected athlete. In response to this evolving public interest, media coverage of injuries to MLB players has also increased, with the most attention focused on upper extremity injuries in pitchers. This focus on upper extremity injuries in pitchers is not surprising, given the fact that throwing injuries among pitchers are highly prevalent at all levels of the sport. Moreover, these injuries in MLB pitchers have serious professional and financial implications for both the players and the league, with reported losses of approximately 500 million dollars annually in the MLB because of injuries to professional pitchers alone.^2^ Among professional players, upper extremity injuries in pitchers account for nearly 50% of all injuries in MLB and result in an average of 460,432 days on the MLB disabled list (DL).^2^

Interestingly, the increasing media coverage of MLB injuries has been mirrored by an increasing rate of MLB injuries. Multiple previous studies of have consistently reported a concerning trend of increasing injury rates among MLB players over recent years, despite improvements in training, diagnostic modalities, conditioning, and surgical techniques.^2-5^ From 2011 to 2016, a reported 45,123 nonseason ending injuries occurred, resulting in a total of 722,176 days missed from play.^6^ In general, overhead athletes are particularly susceptible to chronic overuse injuries, and these risks are only amplified in the setting of the grueling and extensive regular season seen at the major league level.

The interplay of increased public demand for access to MLB injury information and the mainstream media's increased focus on this topic has given rise to a plethora of publicly available MLB injury databases, including team websites, media releases, and baseball reporting sources. As a result, several studies have been published in the literature using publicly available player data to assess changes in player performance and RTP because of injuries in MLB players. In addition, further studies have used the MLB Health and Injury Tracking System (HITS), a centralized, deidentified medical database, developed to longitudinally track medical histories. With the implementation of a league-wide database, and access to publicly available player data, information on injury outcomes, associated risk factors for specific injuries, and guidelines for successful RTP have become more widely available and are currently used to guide treatment decisions on a daily basis.^7^

Despite the abundance of media attention and medical literature focusing on upper extremity injuries among MLB pitchers, there have been relatively few investigations that have explored the epidemiology of core and lower extremity injuries in this athletic population. Moreover, the concept of the “kinetic chain” has gained popularity in recent years, and growing evidence exists within the medical literature, suggesting that deficits in any component of this chain can adversely affect performance and potentially increase the risk for injuries in the upper extremity. This evolving concept supports the need for a more generalized approach to the elite overhead athlete, with increased focus on strength and kinematic deficiencies in the lower extremities, hips, core, and entire upper body, rather than exclusively focusing on the shoulder and elbow in isolation.^8,9^ However, outside of the known upper extremity and throwing-related injuries, awareness and knowledge of the variety of other pathologies experienced by MLB athletes is lacking. As such, the primary goal of this systematic review is to summarize all data published on truncal and lower extremity orthopaedic injuries among professional baseball players in the MLB. In addition, the current literature will be stratified according to the level of evidence and primary source for injury data, with comparisons made to identify differences observed between investigations using different data sources.

## Methods

A comprehensive search of publications identified studies reporting lower extremity, core, or spinal orthopaedic injuries in professional baseball players at the major league level. The search strategy was conducted using the Embase, Medline, and PubMed databases. The Preferred Reporting Items for Systematic Reviews and Meta-Analyses guidelines were used during the review process.^10^ Search terms including “Major League Baseball,” “MLB,” “professional baseball,” “professional baseball players,” and “injury” were used to maximize the sensitivity of the search. Inclusion criteria consisted of original studies, studies with players reporting orthopaedic injuries, and descriptive epidemiological studies of injuries in MLB players. Exclusion criteria included case reports, non-English language studies, reviews or editorials, non-MLB studies, studies reporting only injuries in Minor League Baseball players, and studies of nonorthopaedic injuries, concussion injuries, facial injuries, and vascular injuries. No limits were placed on the level of evidence or timing of the studies—the search period was from January 1980 to August 2019. The articles were divided into eight injury categories based on anatomic site, including spine, trunk, hip and pelvis, thigh, knee, foot, ankle, and general/other. The following data were extracted from each study: publication year, study design, the level of evidence, the source of data, study duration, total players, the number of injuries, player demographic information, recovery time, RTP or sport, return to the previous level of competition, mean career length after return to sport (RTS), and playing position. For studies combining MLB and Minor League Baseball players, only MLB-specific data were included for the final review. Studies were compared by data source, including the MLB HITS and publicly available information on the internet—including the MLB DL, MLB Transaction Database, and other MLB databases. The DL is a method used by MLB for teams to remove injured players from the active 26-player roster to allow the addition of healthy players from constituent minor league teams, farm teams, free agency, players now recovered from injury, or recently traded players. Despite removal, players remain on the overall 40-player roster, and depending on the severity of injury, players could be placed on 10-, 15- or 60-day injured list.^11^ Other sources of data were acquired from team medical staff, player medical records, and other databases.

## Results

**Figure 1:**
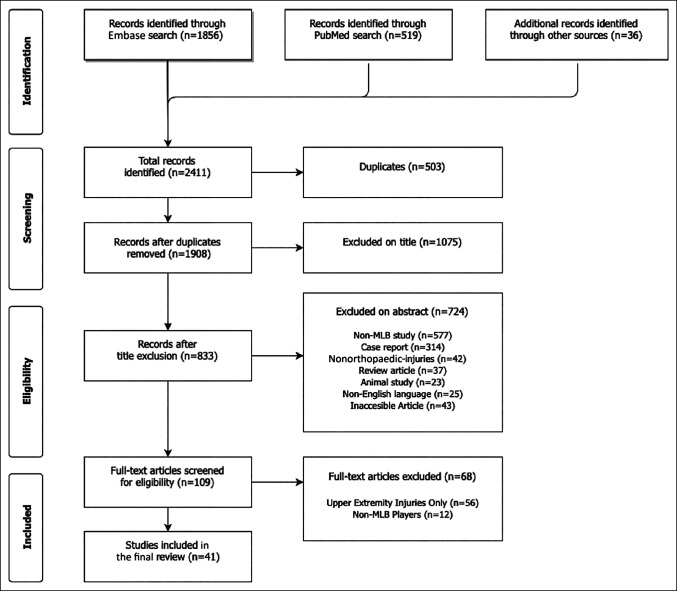
Chart showing the Preferred reporting items for systematic meta-analyses diagram of included articles.

**Figure 2:**
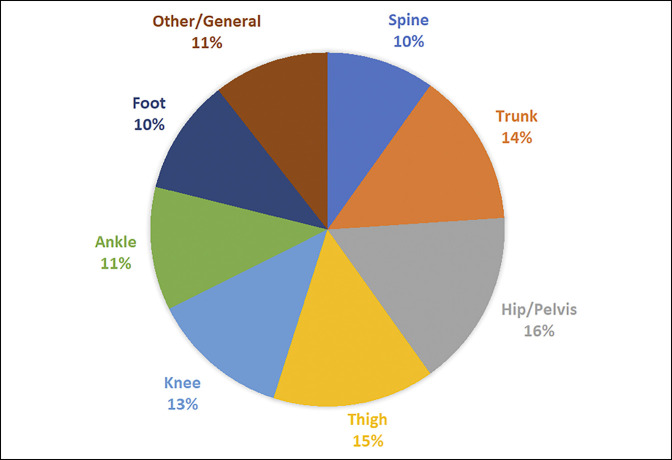
Chart showing the percentage of articles by anatomic location of orthopaedic injury. Injury categories are not mutually exclusive.

**Figure 3:**
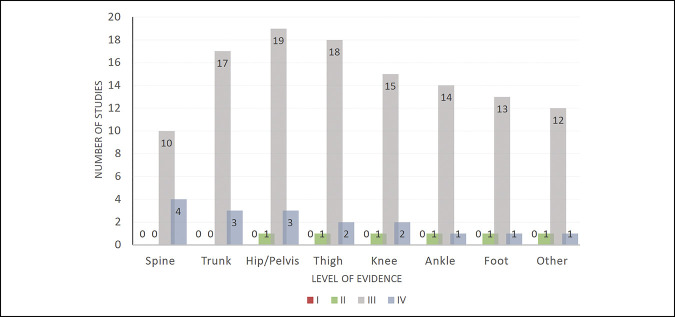
Chart showing the number of articles by the level of evidence and anatomic location of orthopaedic injury. Injury categories are not mutually exclusive.

The initial query yielded a total of 2,411 articles, of which 503 duplicates were removed. The remaining 1,908 articles were screened by title and abstract, and 1,799 articles were excluded because of failure to meet one or more study inclusion criteria. The manuscripts of the remaining 109 articles were reviewed further, and 68 were excluded for failing to meet additional inclusion criteria. A total of 41 studies, with 1,539 total study participants, were included in the final analysis (Figure 1). Each article was assigned to one of seven categories based on the anatomic site of orthopaedic injury. The most common sites of injury were hip and pelvis (16% of studies), followed by thigh (15%), trunk-related (14%), and knee (13%) injuries. (Figure 2). Articles were compared by the level of evidence and stratified by injury site, as shown in Figure 3. Most articles were level III evidence (83.7% of studies), followed by level IV (12.1%), and level II (4.2%). No level I studies were observed. Articles were also compared by data source and type of injury, as shown in Table 1. The most common data source was the internet (29.8% of studies), which includes the MLB DL (25.5%), MLB Transactions Database (11.1%), and other MLB databases (0.85%). Other data sources included the MLB HITS (22.1%), team medical staff (5.5%), player medical records (2.1%), single institution/surgeon (1.3%), and data consultant (1.7%). Data sources were not mutually exclusive.

**Table 1:** The Number of Articles by Source of Data and Anatomic Location of Injury

Injury Type	MLB Health/Injury Tracking System	Internet-based Search of Publicly Available Data	MLB-Designated Lust	MLB Transactions Database	Other MLB Databases	Team Medical Staff	Player Medical Records	Single Surgeon	Data Consultant/Service	Team Doctor Survey	Player Survey
Spine	4	8	8	4	1	0	1	1	1	0	0
Trunk	8	7	6	1	0	4	2	0	1	0	0
Hip/pelvis	7	11	10	4	1	2	0	2	1	0	0
Thigh	8	10	9	4	0	3	1	0	0	0	0
Knee	6	10	8	4	0	2	1	0	0	0	0
Ankle	6	9	7	3	0	1	0	0	0	0	0
Foot	6	8	6	3	0	1	0	0	0	0	0
Other/general	7	7	6	3	0	0	0	0	1	0	0

Data sources and injury categories are not mutually exclusive. ‟Internet” includes publicly available media sources and team reports of injuries and player transactions. ‟Medical records” refers to studies whose authors or team medical staff had access to players' medical records. ‟Data consultant” includes private or commercial third-party data analysts or websites providing player data, information, and injury and team reports.

### General Epidemiologic Trends of Major League Baseball Injury

Injury rates in MLB players have reportedly increased over time with higher rates found in April (5.73/1,000 exposures), whereas the lowest rates of injury are found in September (0.54/1,000 exposures).^4^ Between 2002 and 2008, players were designated to the DL at a rate of 3.61 per 1,000 athlete exposures (AE). Most injuries (51.4%) affected the upper extremity, although spine and core-related injuries were also common, accounting for 11.7% of all reported injuries.^4^

Pitchers sustained a higher injury rate than fielders (4.16 per 1,000 AE versus 3.10 per 1,000 AE), and pitchers made up a larger proportion of days missed on the DL compared with fielders.^4^ Investigations on pitcher workload and subsequent risk of injury showed no notable association between cumulative total innings pitched (IP), starts, pitch counts or pitches per start, and pitcher placement on the DL for any musculoskeletal reason or upper extremity reason.^12,13^

Regarding injuries sustained by nonpitchers, sliding was found to be the most common mechanism of injury, accounting for 12.3% of the 236 injuries that required surgical intervention over the 5-year period between 2011 and 2015.^14^ In 2015, 53% of sliding-related injuries resulted from head-first slides with most occurring at second base.^14^

Overall, 2.9% of players hit by pitch, resulting in injury with a 31% of injuries affecting the head and neck.^15^ Increasing pitch velocity was associated with a linear increase in both injury rate and days missed.^15^ Players hit by pitch spent an average of 11.7 days on the DL per injury, with 3.1% of these injuries requiring surgical intervention.

Injury rates for catchers was 2.75 per 1,000 AE between 2001 and 2010, with most injuries resulting from noncollision mechanisms.^16^ Among catchers, the most common site of injury from all causes was the leg. Among injuries to catchers that resulted from collision-specific mechanisms, the most common injury site was the knee. Catchers, overall, missed an average of 50.8 days for all injuries across a 10-year period.^16^

### Spinal Injuries

Spinal injury was found to be a notable cause of player DL designations, with lumbar and cervical disk herniations accounting for most spinal injuries reported.^3^ Previous studies found baseball players to be at high risk of lumbar disk degeneration, with a rate >3 times that of nonathlete control subjects.^17^ Despite this, few studies have investigated the outcomes and the potential for RTP after lumbar disk herniation. In one of the earliest studies on spinal pathology in MLB players, Roberts et al^18^ reported on 11 pitchers with cervical disk herniation (CDH) and 29 with lumbar disk herniation between 1984 and 2009. Among the 11 players diagnosed with a CDH, 73% were treated with either ACDF or cervical disk replacement. Among pitchers, 73% with CDH returned to play at an average of 11.6 months. Those treated with surgery returned at a higher rate compared with nonsurgically managed players (88% versus 33%); however, this difference did not reach significance. Furthermore, no notable differences in age or posttreatment career length were found. Among 29 players diagnosed with lumbar disk herniation, 69% underwent microdiscectomy and/or laminotomy.^18^ All MLB pitchers suffering lumbar disk herniation ultimately returned to play at an average of 7.3 months. Further analyses showed surgical intervention to be more common in older and more experienced players compared with players managed nonsurgically (*P* < 0.03), yet no statistically significant differences were noted in posttreatment career length regardless of the treatment.^18^

In postinjury performance, players with CDH pitched, on average, fewer innings per season on RTP (*P* = 0.04), but no statistically significant differences in postoperative performance were seen when stratified by treatment.^18^ Furthermore, for pitchers with lumbar herniations, surgical intervention was associated with fewer innings pitched (*P* = 0.02) and an increase in walks plus hits per inning pitched (WHIP) (*P* = 0.02) postopertively.^18^

In a 2011 study evaluating 342 professional athletes, Hsu^19^ reported on outcomes of 68 baseball players with lumbar disk herniations. Compared with the other sport, MLB athletes had a significantly greater rate of RTP after suffering a lumbar disk herniation (*P* < 0.05). No notable differences in outcomes or RTP were found when these players were stratified by treatment type; however, players undergoing diskectomy ultimately played markedly less games and total years compared with the nonsurgical cohort.^20^

More recently, Watkins et al^21^ performed a review of 26 professional athletes undergoing anterior cervical diskectomy and fusion (ACDF), which included 5 MLB players. Within this study cohort, five patients were unable to RTP postoperatively, one of whom was an MLB player.^21^

Posterior foraminotomy (PF) and total disk arthroplasty are also potential alternatives to ACDF. In a 2018 analysis, Mai et al^22^ reported on the outcomes of 101 professional athletes, including 21 MLB players, who were treated for symptomatic CDH. The authors found that 81% of these patients underwent ACDF and 9.5% had a PF, reporting higher RTP rates and shorter time to RTP after PF compared with ACDF.^22^ In addition, increasing age was found to be a notable negative predictor for RTP, regardless of the type of surgical intervention. In postoperative performance, the study by Mai et al reported improved performance in baseball players—compared with other sports—after surgery; however, no differences were found between surgical options. Notably, all pitchers were able to RTP, but notably, three of eight starting pitchers returned in a relief role.^22^

### Trunk/Core Injuries

The single season epidemiological study of musculoskeletal injuries in professional baseball players Li et al^23^ found that abdominal and groin injuries led to 5.6 and 6.4 times the rate of days missed for pitchers compared with both position players and all players, respectively. A subsequent review of the MLB DL focusing on core injuries—such as oblique strains, abdominal strains, rib cage/rib muscle strains, intercostal muscle strains, and rectus abdominis strains—over a 20-year period identified 393 abdominal injuries (92% internal/external oblique, intercostal, or rib muscle; 1% rectus abdominis strains; and 7% abdominal muscle strains) with 44% occurring in pitchers.^24^ Since 1991, there has been an upward trend in abdominal muscle injuries from the lowest injury rate of 0.0111 per player in 1993 to 0.023 per player in 2007.^24^ In kinematics, deficits in hip internal rotation of 5° have been correlated with core injury (odds ratio, 1.4, *P* = 0.024 for pitchers; odds ratio, 1.35, *P* = 0.026).^25^ From the 1990s to 2000s, a 22% increase was noted in the rate of abdominal injuries, yet the rate has remained relatively constant since 2003.^24^ Average time spent on the DL for abdominal strains was 30.6 days with pitchers averaging 35.4 days compared with 26.7 for position players (*P* < 0.1). Recurrent injuries were found in 12.1% of players (33% in pitchers and 66% position players), and 54.8% of injuries were sustained either during the same season or the following season.^24^ In pitchers, 78.1% of injuries occurred contralateral to the dominant arm compared with 70.3% of injuries sustained contralateral to the dominant batting side in position players.^24^ Laterality was associated with recovery because positional players missed significantly more time for contralateral injuries as compared to ipsilateral injuries (28.9 versus 21.2 days, *P* = 0.03), whereas pitchers missed more time for ipsilateral injuries as compared to contralateral injuries (44.5 versus 32.8 days, *P* = 0.04).^24^

Within the MLB HITS medical record system, 1,515 abdominal wall or muscle injuries were recorded between 2011 and 2015.^26^ Specifically, 1,075 were oblique injuries and 79 were season-ending injuries. Of the remaining 996 oblique injuries, 26% resulted in DL designations and accounted for a total of 6,132 missed days by MLB players.^26^ Players missed a mean 23.7 days with a recurrence rate of 10.48%. The calculated injury rate was one oblique strain for every 93.8 games played or one injury for every 1,342 appearances.^26^ Overall, the annual incidence decreased over time in MLB (*P* = 0.037) with a mean of 199.2 injures per year.^24,26^

During a similar period (2010 to 2015), Conte et al^24^ documented 35 abdominal injuries among MLB players, reporting that 37.1% of these injuries occurred in pitchers.^25^ The most frequent abdominal injuries included intercostal strains (46%), rectus strains (23%), and oblique strains (15%) for pitchers, whereas intercostal strains (55%) and oblique strains (23%) were more common among hitters.^25^ Relating hip range of motion (ROM) with injury risk found no notable trend between decreased hip internal rotation and abdominal injury in pitchers.^25^

More recently, a retrospective review of MLB pitchers placed on the DL during the 2014 to 2015 season identified 330 MLB pitchers with 454 injuries.^27^ Core and hip/groin injuries represented 14% of all injuries.^27^ Most core injuries occurred at the start of the season in April and at the end of season the after August. Three pitchers ultimately required surgery, resulting in a mean 164 days on the DL, yet no notable preoperative factors were observed for pitchers requiring surgery compared with those treated nonsurgically. Thirty-three percent of these core injuries occurred on the ipsilateral side of the throwing arm.^27^ Most injuries (57%) occurred in starting pitchers and of those pitchers with core injuries, 79% were able to return from the DL in the same season at an average of 47 days.^27^ Recurrence occurred in only 6% of pitchers.^27^

In terms of player performance, pitchers with core injuries threw fewer innings per game (4.96 versus 5.3, *P* = 0.031) but more pitchers per game (85.5 versus 78.4, *P* = 0.026) the year of injury compared with the rest of their career.^27^ Overall, pitchers experienced a notable decrease in fastball velocity the year of injury compared with their overall career (91.6 versus 92.2 mph, *P* = 0.001), with starters showing a similar decline (91.3 versus 91.9 mph, *P* = 0.001), whereas relief pitchers saw no change in velocity.^27^ Hitters with core injuries had decreased home runs per nine innings (0.95 versus 1.07, *P* = 0.023) and strikeouts per nine innings (6.94 versus 7.63 strike outs, *P* = 0.020).^27^

### Hip and Pelvis Injuries

Previous literature has reported on the association between hip motion and hip, groin, and hamstring injury.^28^ Li et al^28^ found that in patients with differences in hip internal rotation and total arc motion, in-season injuries were more likely in younger players (*P* < 0.05), positional players, and in players with a history of hip, hamstring or groin injury (*P* < 0.001). Within the MLB Injury database, Coleman et al^29^ identified hip and groin injuries in 17% of professional baseball players between 2011 and 2014. Infielders experienced the largest proportion of hip and groin injuries, and most were the result of noncontact injuries that occurred while fielding.

Marshall et al^27^ investigated MLB pitchers in 2014 to 2015 and found that 7% MLB pitchers sustained hip/groin injuries, with the highest rate of injury occurring at the beginning of the season. The authors reported that 57% of hip/groin injuries were ipsilateral to pitchers' throwing arm, and 53% of hip/groin injuries occurred in starting pitchers.^27^ Pitchers were able to return in the same season of injury 73% of the time and spent an average of 37.7 days on the DL.^27^ Recurrent injuries occurred in 56% of players, and 10% of these players with recurrent injury were subsequently able to return to competitive play.^27^ Pitcher-specific characteristics, such pitch choice, were found to differ during the year of injury as compared to injury-free seasons. The authors report a decrease in the percentage of fastballs thrown (54.4% versus 57.3%, *P* = 0.039) and an increase in the percentage of curveballs thrown (17.2% versus 12.0%, *P* = 0.009) in the season of injury as compared to individual career pitch breakdowns.^27^ Pitchers showed no change in innings per game, pitches per game, or pitches per inning in the year of injury compared with the remainder of their careers.^27^ Moreover, starters ultimately experienced a notable decrease in fastball velocity during the year of injury compared with the rest of their career (90.5 versus 91.3 mph, *P* = 0.005). For hitters, hip and groin injuries resulted in an increase in strikeouts per nine innings (7.87 versus 7.51, *P* = 0.016), but no differences in walks, earned run average (ERA), or wins above replacement (WAR) were observed during the year of injury.^27^

Regarding femoroacetabular impingement (FAI), Frangioamore et al^30^ reported on 51 MLB players who underwent hip arthroscopy for symptomatic FAI between 2000 and 2015. Among the 51 players in their study cohort, 82.4% had labral repairs, 86.3% underwent cam osteoplasty, 80% had acetabular osteoplasty, and 69% had excess capsular tissue requiring either capsular plication or thermal shrinkage.^30^ The authors reported excellent RTP after surgery, with 95% of players returning to professional baseball postoperatively.^30^ After surgery, the average playing career was 3.6 ± 2.9 seasons with no notable differences in games played in the season of RTP compared with the season before injury.^30^ In addition, no differences were seen in postoperative playing career when stratified by player position.^30^ In pitchers, 71% (17 of 24) underwent hip arthroscopy procedures involving the lead leg of the pitching stance, whereas 74% (20 of 27) hitters had surgery performed on the lead leg of their batting stance. Among all players, no notable differences were found in postoperative ERA or batting average compared with preoperative baselines in pitchers or hitters.^30^

A more recent study by Schallmo et al^31^ reviewed the outcomes of hip arthroscopy procedures performed between 1999 and 2016, reporting a 84.6% and 78.8% RTP rate for pitchers and hitters, respectively. Regarding laterality, 83.3% of pitchers who had surgery on their lead leg returned to play, whereas 70.6% of hitters undergoing surgery on their lead leg were able to return to competition.^31^ Compared with other sport, MLB athletes were significantly older at the time of surgery (*P* = 0.022) and had shorter preoperative career games and years played at the time of surgical intervention (*P* = 0.029 and *P* = 0.003, respectively). Younger age (*P* = 0.022), shorter preoperative careers, in years, and a greater amount of games played in the season before injury were statistically significant factors associated with successful to RTP.^31^ Ultimately, MLB players competed for 2.8 ± 2.6 years or 192 ± 257 games postoperatively.^31^ Compared with other professional athletes, MLB players missed the fewest games postoperatively before returning to play (*P* = 0.004); however, MLB players had the lowest median career survival (3.5 years) after the Kaplan-Meier survivorship analysis.^31^

In performance measures, overall no statistically significant differences were noted in performance scores (WHIP for pitchers, on-base plus slugging [OPS] for hitters) postoperatively.^31^ A subgroup analysis revealed hitters undergoing surgery on their lead leg demonstrated a significant decrease in OPS (*P* = 0.041) in their first postoperative season; however, they were able to recover to their preoperative metrics by postoperative seasons two and three.^31^ Although no notable difference was seen in performance among pitchers undergoing hip arthroscopy, additional analyses revealed that MLB pitchers had significantly decreased average four-seam fastball velocity during the first postoperative season (*P* < 0.001).^31^ Similar reductions in velocity were found specifically when looking at MLB pitchers who had surgery on their lead leg (*P* = 0.004), and a significantly decreased pitch count in the first postoperative season (*P* = 0.007) compared with the season before injury.^31^ However, both metrics returned to baseline preoperative numbers by postoperative seasons two and three.^31^

A similar, more recent, study on performance and RTP after FAI reported 82.5% of players returning to sport at a mean of 8.3 ± 4.1 months postoperatively with no notable differences in RTS rates when compared between positions.^32^ Regarding surgical extremity, similar RTS rates were seen in pitchers regardless of the laterality of the surgical leg (81.3% for back leg versus 76.9 for lead leg). At 1-year postoperatively, the average MLB career survival was 78.9%.^32^ In performance, no notable differences were seen in plate appearances or innings pitched postoperatively compared with control subjects.^32^ Furthermore, neither hitters nor pitchers had any notable difference in performance postoperatively when compared with preoperative baselines.^32^

### Thigh Injuries

Within baseball-specific sporting activities, hamstring injuries most commonly occur in the process of running bases or lunging/diving for the ball. In their 2010 study evaluating hamstring injuries in elite-athletes, Cooper and Conway^33^ reported on eight MLB players with complete distal semitendinosus tendon ruptures. From this cohort, 87% ultimately had surgery, but 33% initially trialed a course of nonsurgical management. Average recovery after surgery was 12 weeks compared with 7 weeks with nonsurgical management.^33^

Using the MLB HITS, Ahmad et al^34^ prospectively reported a rate of 0.7 hamstring injuries per 1,000 AE across major league players, with most injuries sustained by right-handed batters running to first base. Okoroha et al^35^ similarly looked at trends in hamstring injuries and found an increased rate of injury from one injury per 39 games in 2011 to one injury per 30 games in 2016, with the largest percentage of injuries occurring in April and May. Infielders (37.0%) made up most injuries with >50% of the injuries occurring while running to first base.^35^ Average time to RTP was 14.5 days with significant differences in days missed from grade 2 and 3 hamstring strains compared with grade 1 injuries (*P* = 0.005 and *P* = 0.002, respectively). Recurrence rates reached 16.3% in MLB athletes with increased time lost compared with index injuries (*P* = 0.02).^35^ RTP time was also influenced by the treatment type and age as surgical management, the use of platelet-rich plasma and increasing age were associated with more days lost from play.^35^

In an analysis of MLB pitchers specifically, Howard et al^36^ reported a total of 78 DL designations in 65 pitchers because of hamstring injuries in a 10-year period. The landing leg was most often injured (67.9%), with 93.6% of injuries resulting in stays on the DL of 10 or 15 days.^36^ Median RTS was 18 days with 76.9% of pitchers returning to sport in <30 days and 35.9% returning in <15 days.^36^ Of note, none of the aforementioned studies were able to make comparisons on RTP based on the tear location.

### Knee Injuries

#### Anterior Cruciate Ligament Tears

Mai et al^37^ investigated player performance after anterior cruciate ligament injuries in professional athletes. For MLB players specifically, 21 athletes sustained anterior cruciate ligament tears between 1984 and 2013 with 81% returning to play postoperatively.^37^ Of the 21 players, 14% sustained a retear, requiring a revision procedure, and 81% of all injuries occurred in season.^37^ Postoperatively, the average career lasted 2.9 years, with 81% of injured players remaining on an active roster three seasons after injury, but no notable changes in performance were seen across batters and pitchers within the first three seasons after surgery.^37^

#### Articular Cartilage Injury

When compared the major professional sport (the National Basketball Association, National Football League, and National Hockey League), after knee microfracture surgery, Schallmo et al^38^ found that MLB players were significantly older at the time of surgery (*P* = 0.001) and had the highest RTS (100%, *P* = 0.013) with an average of 254 ± 133 days missed and an average postoperative career of 3.2 ± 3.5 years. In addition, MLB players were found to have the highest median postoperative career length of 2.8 years.^38^ In performance, MLB pitchers had a decrease in WHIP, whereas batters had a lower OPS compared with the preinjury baselines (*P* = 0.002).^38^

### Foot and Ankle Injuries

In their 2018 case-control study, Saltzmann et al^39^ reported an increasing annual incidence of Achilles tendon injuries among MLB players, with 20% of these injuries occurring during the preseason or first month of the regular season. Most (62%) were able to return to major league play for at least 81 games after surgical repair, regardless of the differences in player age, body mass index, the side of involvement, or MLB experience before injury.^39^ Ultimately, no association was found between Achilles tendon injury and changes in player statistical data postoperatively.^39^ Compared with control subjects, injuries to the power-limb were associated with playing 28.65 fewer games (*P* = 0.039), 141.63 fewer plate appearances (*P* = 0.010), 1.44 lower speed scores (*P* = 0.004), and 0.76 fewer stolen bases and caught steal runs above average (*P* = 0.021).^39^ Similar results were found in the evaluation by Trofa et al^40^ of RTP and performance after surgical repair of Achilles tendon ruptures. In the study, five MLB players underwent surgical intervention at an average age of 31.3 years. Postoperatively, all MLB players were able to return to competitive play with no notable differences in games played, or performance.^40^

### Comparison of Overlapping Findings From MLB Health and Injury Tracking System Versus Medical Records Versus Internet-Based Studies

#### Cervical Disk Herniation

Both Mai et al^22^ and Watkins et al^21^ focused on the outcomes after the management of cervical disk herniations with data obtained from publicly available internet-based resources and individual case series, respectively. Mai et al^22^ identified 21 MLB players with cervical disk herniations, for which 17 underwent ACDF, two underwent PF, and two had a total disk arthroplasty. Alternatively, Watkins et al^21^ reported a case series of 5 MLB players who underwent ACDF. Both studies reported that all players were cleared for a RTP after ACDF, but only 37.5% of starting pitchers returned to relief roles in the cohort reported by Mai et al.^21,22^ However, MLB players had a notable decrease in performance (WHIP) after any cervical surgery, yet no differences regardless of whether either ACDF or PF were performed.^22^ Overall, both studies examined pathology across several professional sport, with a relatively smaller patient sample of MLB players who might have prevented further analyses. No studies were currently available that used the MLB HITS database to evaluate spinal pathology.

#### Femoroacetabular Impingement

A single study was identified that used the MLB HITS to examine hip and groin injuries in professional baseball players. This study, however, did not report specific pathologies or diagnoses associated with each injury, making comparisons to other studies in the literature difficult.^29^ Rather, comparisons were more readily made between studies using publicly available internet-based resources and those involving individual team medical records.^30-32^ Jack et al^32^ used publicly available data to identify 50 players between 2000 and 2017 undergoing 57 hip arthroscopy procedures for FAI with a RTP of 82.5% at 8.3 months and no difference in RTP rates by position. Thirty-one surgeries were performed on pitchers with a RTP of 77.4%, whereas nonpitchers had a rate of 88.4%. Furthermore, no difference was seen in innings played or plate appearances postoperatively.^32^

Compared with studies using individual team medical records, Schallmo et al^31^ reported on 47 players from 1999 to 2016, whereas Frangiamore et al^30^ identified 44 players (51 hip arthroscopies total) from 2000 to 2015. Overall, the RTP rates of 81.2% and 95% were reported. By position, pitchers returned at a rate of 84.6% and 95.8% in the investigations by Schallmo and Frangiamore, respectively, whereas nonhitters returned at 78.8% and 96.3%, respectively.^29,30^ Postoperative career lengths varied from 2.8 seasons^31^ and 3.6 seasons,^31^ yet both studies note no changes in performance measures comparing preinjury and postoperative statistical data.^30,31^ A similar number of injuries were reported over generally comparable time periods showing some consistencies between what is reported in the press and on the internet compared with individual medical team records; however, a discrepancy in RTP rates exists based on the data source used.^30-32^

#### Hamstring Injuries

Studies focusing on hamstring injuries in the MLB have mostly used the HITS database with only two other studies using internet-based data and medical records, respectively.^33-35^ In 2011, Ahmad et al^34^ identified 50 hamstring strains for an injury rate of 0.7 per 1,000 AEs. Okoroha identified 441 injuries between 2011 and 2016 with an injury rate of 1.09 AE with 3.4% season-ending injuries and over half of those injuries resulting in more than 7 days lost.^35^ Overall, the RTP rates were increased with surgical treatment.^35^ In the same study, 20% (n = 89) of injuries occurred in pitchers.^35^ Comparatively, Cooper and Conway^34^ reported a case series that included eight hamstring injuries in MLB players over a 14-year period, treated by two different surgeons. Using internet-based resources, Howard et al^36^ analyzed the outcomes of 65 pitchers who sustained hamstring injuries requiring placement on the DL between 2008 and 2017.

Given the small number of studies dedicated to hamstring injuries, and varied scopes of each investigation, gross comparisons between data sources are limited. The study by Cooper and Conway^33^, for example, contained less data specific to the MLB, whereas the study by Howard et al^36^ focused specifically on pitchers. The MLB HITs database, however, seems to provide a more comprehensive injury rate for hamstring injuries, and time lost.^34,35^ Furthermore, more injuries were detected over a shorter period among pitchers in the investigation performed by Okoroha et al, highlighting the potential for missed injuries in internet-based studies.^35,36^

#### Abdominal Strains

Using data from MLB HITS database between 2010 and 2015, Camp et al^26^ reported an injury rate of one oblique strain per 1,342 appearances among MLB players, with a decreasing trend in overall abdominal injury rate over the same study period. Across MLB players, 259 sustained an oblique injury with a total of 6,132 total days missed and a 10.48% reinjury rate.^26^ More comprehensive studies have been performed using internet-based resources.^24,27^ Conte et al^24^ identified 393 abdominal muscle strains, for which 44% were in pitchers from 1991 to 2010.^24,26^ Oblique injuries, intercostal injuries, or rib muscle injuries occurred in 92% of cases with the remainder consisting of 7% general abdominal muscle strains and 1% rectus abdominis strains.^24,26^ Conte found an upward trend in abdominal muscle strains over the study period in contrast to the investigation by Camp et al; however, from 2003 to 2010, the injury rate was relatively stable.^24,26^ The reinjury rate was 12.1%, higher than reported from HITS database data, with an average of 30.6 days spent on the DL for all players.^24,26^ Pitchers spent more time on the DL (35.4 days) compared with position players (26.7) with an increasing trend in days spent on the DL over the study period.^24^ Over a 1-year period (2014 to 2015), Marshall et al^27^ used publicly available player data to identify 330 MLB pitchers with 63 core injuries, with 47.6% affecting the obliques, abdominal muscles, or intercostals. Comparatively, Marshall notes a lower reinjury rate of 6% over a single season with an average of 47 days spent on the DL for all pitchers.^27^ No studies using individual player medical records specifically evaluated abdominal strains or injuries.

## Discussion

This systemic review provides a comprehensive summary of the orthopaedic literature focusing on lower extremity and truncal injuries among MLB players. Although robust historical literature reporting on upper extremity injuries among MLB players exists, these studies have often focused on pitchers and have rarely provided insight into the epidemiology of orthopaedic injuries affecting the core and lower extremities in MLB players of all positions. With the establishment of the MLB HITS and the recent proliferation of publicly available MLB injury databases, however, there has been an increase in publications reporting on the epidemiology, treatment, and outcomes of truncal and lower extremity injuries in the MLB.

The findings of this study reveal most of this literature to be level III evidence (83.7%) and the most common source of data to be publicly available MLB injury databases, followed by the MLB HITS database. Among the studies included in this review, the hip and pelvis were the most commonly reported anatomic site of injury (16% of studies), followed by thigh (15%), trunk-related (14%), and knee (13%) injuries. Interestingly, the highest incidence rate for injuries of all types was found to occur in the first month of each season and decline markedly as the season progressed, suggesting that off-season deconditioning may be a major predisposing factor to injuries of all types in MLB players.^4^ Excluding pitchers, the most common mechanism of injury was found to be sliding, with impact injuries, such as being hit by a pitch or colliding with another player, accounting for only a minority of reported injuries.^14,15^

Injuries to the hip and groin were more commonly seen in infielders through noncontact mechanism.^29^ Among pitchers, hip and groin injuries most often occurred toward the beginning of the season, with a subsequent same-season RTP rate of 73% without resultant changes in pitching performance measures for the remainder of their careers.^27^ In players suffering from FAI, labral repair and osteoplasty of cam or acetabular lesions were the most commonly performed procedures.^30^ For both pitchers and hitters, the lead leg was most often affected by symptomatic FAI-requiring surgery.^30^ Ultimately, RTP rates ranged from 70.6% to 96% postoperatively with younger age, shorter preoperative career lengths, and greater amount of games played, in the season before injury, markedly associated with successful RTP.^30-32^ Furthermore, current literature has grossly found no statistically significant differences in performance after surgery, regardless of the position.^30-32^ Compared with players with FAI in other professional sport, MLB players with FAI had the lowest median career survival and played fewer games postoperatively.^31^

Hamstring injuries are a common injury within the MLB most often occurring during base running or when fielding.^34^ Similar to hip and groin injuries, the largest percentage of hamstring injuries occurred toward the beginning of the season with batters running to first base composing most all injured players.^34,35^ In RTP, higher grade injuries led to markedly more days missed from competitive play with recurrences further amplifying time lost from play.^35,36^ Finally, based on the current literature, factors associated with prolonged RTP included surgical intervention, the use of platelet-rich plasma, and older age.^35^

Trunk and core-related injuries have become an increasingly common injury, with a rising trend in abdominal injuries in the 1990s, yet more recent literature has shown a steady decline in more recent years.^24,26^ In concert with what has been seen with aforementioned injuries to the hip, groin, and thigh, core-related injuries most often occur at the beginning of the season but a fair number of injuries also seem to arise toward the end of the regular season as well.^27^ For both pitchers and hitters, injuries predominately affected the side contralateral to either the dominant arm or batting side, respectively.^24^ Exact proportions of injury types are variable, depending on the source of injury data, but based on the current literature, most trunk and core injuries have been related to oblique or intercostal strains with time lost ranging from 23.7 to 30.6 days on the DL.^24,26^ Overall, pitchers, on average, were found to miss more time from play (35.4 to 47 days) with those undergoing surgery spending more time on the DL.^24,27^

To our knowledge, this is the most comprehensive review of current literature on truncal and lower extremity injuries in MLB. As such, this review provides core knowledge that can be used for treating physicians and medical staff for professional baseball teams that reflects the current frequencies, outcomes, and risk factors for core, lower extremity. and spinal injuries often found in MLB athletes. There are several limitations to note, however. The MLB HITS database was used for several reviewed studies, but it must be mentioned that this database has been used in the literature to compile injury data from both Minor League Baseball and MLB.^7^ Only findings specific to MLB players were analyzed and presented in this review. Furthermore, as the data reported by the constituent articles within this review was largely variable and heterogeneous, the authors have refrained from making definitive claims about player performance after injury because this is review focused on the epidemiology of injury in MLB and these comparisons are outside the scope of the study. In addition, most studies in this review obtained data from an internet-based search of publicly available data (ie, injury reports, player profiles, and press releases). Inherent to this study design, these studies could not be standardized and are at risk of producing unreliable and less generalizable results. Furthermore, limitations to the data obtained from publicly available reports exist because they lack granular data that are typically seen in medical records. Despite the aforementioned limitations, this systematic review provides orthopaedic surgeons evaluating, and treating, baseball players at all levels with a comprehensive review and resource for the current literature available on truncal and lower extremity pathology in MLB players.
